# Radioactive seed brachytherapy for malignant tumors with navigation system and 3D-printing template: a propensity score-matched analysis

**DOI:** 10.1186/s12885-026-16055-6

**Published:** 2026-04-21

**Authors:** Zhe Ji, Haitao Sun, Yuliang Jiang, Bin Qiu, Yi Chen, Mao Li, Jinghong Fan, Junjie Wang

**Affiliations:** Department of Radiation Oncology, Peking University Third Hospital, Author Adress 49 North Garden Road, Haidian District, Beijing, 100191 P.R. China

**Keywords:** Navigation system, 3D printing template, Radioactive seed brachytherapy, Accuracy, Efficacy

## Abstract

**Objective:**

To compare accuracy and efficacy between Image Navigation System (INS, an optical tracking system for real-time needle guidance) combined with 3D printing template (3DPT) versus 3DPT alone in radioactive I-125 seed brachytherapy for malignant tumors.

**Methods:**

157 patients who underwent CT-guided brachytherapy from July 2020 to June 2023 were included. 27 cases using INS+3DPT were matched 1:1 with cases using 3DPT alone based on propensity score-matching. Needle path error, dose accuracy, and clinical outcomes were compared. Results: Mean needle errors were comparable between INS+3DPT and 3DPT groups: angle error (0.5° vs. 0.5°), depth error (3.5 mm vs. 4.4 mm), and tip error (3.1 mm vs. 2.9 mm). No significant differences were found in dosimetric parameters (D90, V100, V150, V200, CI, EI, HI) or clinical outcomes. The 2-year local control rates were 35.6% and 49.2%, while 2-year overall survival rates were 45.1% and 34.8% for INS+3DPT and 3DPT groups respectively (*p* > 0.05). Conclusion: INS+3DPT showed no significant advantages over 3DPT alone. The use of 3DPT without INS can achieve satisfactory therapeutic quality in current clinical practice.

## Introduction

RSBT (Radioactive Iodine-125 Seeds Brachytherapy) was initially used for the treatment of early-stage prostate cancer [1], and now it is increasingly used for local treatment of various solid tumors in the body [2]. However, as it involves puncture operation, which largely depends on the physician’s personal clinical experience and operational level, the quality of the operation has limited the further promotion and application of RSBT in clinical practice. The application of 3D printing template (3DPT) technology achieves accurate control of seed needles and greatly improves the completeness of preoperative planning [3]. The image navigation system (INS) is an optical tracking system that provides real-time guidance during needle insertion procedures. Combining INS with 3DPT can also obtain good puncture accuracy [4]. This study intends to evaluate the difference in treatment quality between the application of INS combined with 3DPT and the simple use of 3DPT in RSBT, and provide the reference for optimizing treatment schemes and selecting appropriate treatment techniques.

## Methods

### General clinical information

**Fig. 1:**
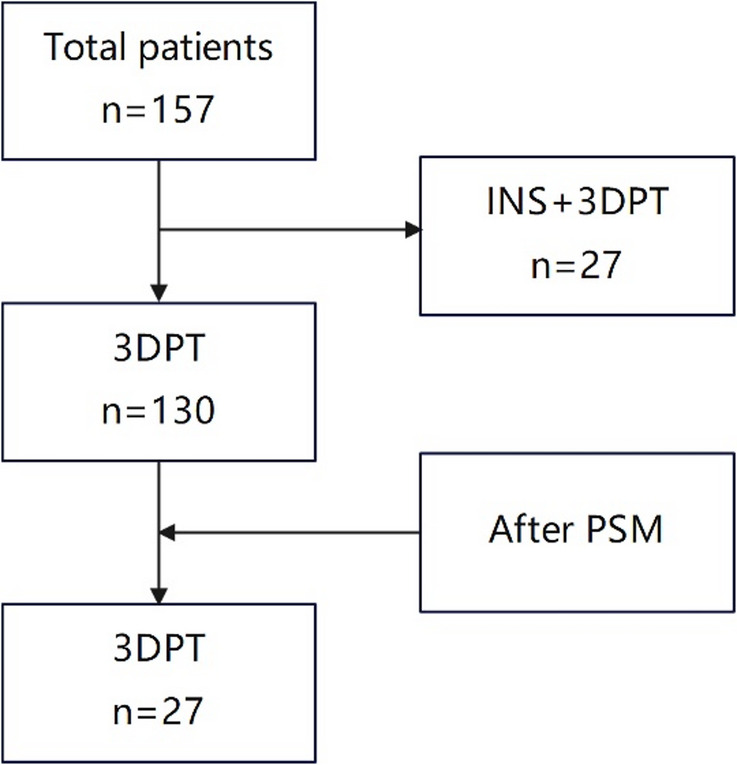
Patient Screening Process: A total of 157 patients were included in the study, with 27 patients in the INS + 3DPT group and 130 patients in the 3DPT-only group. Using the PSM method, 27 cases with characteristics similar to those in the INS+3DPT group were selected from the 3DPT-only group. (INS: Image Navigation System; 3DPT: 3D Printing Template; PSM: Propensity Score Matching)

A total of 27 patients with malignant tumors who received RSBT under INS and 3DPT assist and CT guidance from July 2020 to June 2021 in our department were included. A total of 130 patients who received 3DPT-assisted CT-guided RSBT without INS in our department from July 2021 to June 2023 were selected as matched patients. The matching method employed was propensity score-matching. The matching characteristics included gender, age, KPS, treatment site, and lesion volume. All patients had complete imaging data, preoperative/postoperative plans, and intraoperative operation data. All cases were evaluated and approved for RSBT through multidisciplinary team discussions, considering their complex therapeutic strategies and anatomical characteristics. All treated patients met the criteria for RSBT indications [2]: (1) recurrence after surgery or external radiotherapy, or refusal of surgery or external radiotherapy, with a tumor diameter ≤ 7 cm; (2) definite pathological diagnosis; (3) a suitable puncture path; (4) no bleeding tendency or hypercoagulable state; (5) general condition acceptable (KPS > 70 points); (6) expected survival time is more than 3 months. The study protocol has been approved by the Ethics Committee. All patients signed an informed consent form before treatment. Patient screening process are shown in Fig. 1.

### Material and devices information

(1) Brachytherapy treatment planning system: KLSIRPS-3D (Beihang University, Beijing, China). The system follows the dosimetry protocol established in the TG-43 report and updates by the American Association of Physicists in Medicine, which is the standard guideline for brachytherapy dose calculations [5, 6]. (2) CT scanner: Brilliance Bigbore CT scanner (Philips Healthcare, Amsterdam, Netherlands). (3) INS: IGS-MO optical image navigation system (Xinbo Medical Technology Co., Ltd., Beijing, China). (4) 3D printing system: RS6000 photocuring rapid-prototyping machine (Shanghai Union Technology Co., Ltd., Shanghai, China), with 0.1 mm accuracy. The photocurable resins used meet ISO 10,993 biocompatibility standards [7]. (5) I-125 seeds: Model 6711_1985 (HTA Co., Ltd., Beijing, China), with a half-life of 59.4 days and a dose rate constant of 0.965 cGy/(h·U). (6) Radioactive seed implantation devices: Provided by Mick Radio-Nuclear Instruments Inc. (Mount Vernon, NY, USA) and Eckert & Ziegler BEBIG GmbH (Berlin, Germany).

### Technology flow

#### 3DPT-guided RSBT protocol (in accordance with published experts consensus [8])

The treatment procedure consisted of eight sequential steps: CT simulation, preoperative planning, 3DPT production, 3DPT alignment, fixation needle insertion, needle insertion, seed implantation, and dose validation.

In the CT simulation, patients underwent CT scanning (2.5 mm slice thickness) two days before the procedure. Patient positioning was individualized based on tumor location, with vacuum pad immobilization and surface markers placed to ensure reproducible positioning.

For preoperative planning, CT datasets were transferred to the treatment planning system. The radiation oncologist delineated the gross tumor volume (GTV) and organs at risk (OARs) within a 2 cm margin. The prescribed dose and seed activity were determined, and the distribution and depth of seed needles were designed, including 1–3 fixation needles. The planning system then calculated the required number of seeds and simulated their spatial distribution.

3DPT design and production involved modeling the treatment area in the planning system, incorporating alignment coordinates and needle path information. The 3DPT was manufactured using a 3D photocuring rapid prototyping machine with medical-grade resin material.

For 3DPT alignment, patients were positioned according to the original setup marks. Spinal anesthesia was administered for pelvic procedures, while local infiltration anesthesia was used for other sites. The 3DPT was aligned using surface markers, 3DPT coordinates, and body surface contours.

For fixation needle insertion, fixation needles were inserted under 3DPT guidance, with CT verification confirming accurate positioning.

For needle insertion, the remaining needles were inserted through the 3DPT guide holes to predetermined depths under CT monitoring.

During seed implantation, seeds were implanted according to the preoperative plan, with CT confirmation of seed distribution.

Post-procedure dose validation included CT scanning for dose verification in the planning system, assessing the actual dose delivered to both the target volume and OARs.

#### INS integration protocol

When integrating the INS with 3DPT, the procedure followed the same basic steps, with additional navigation features for enhanced precision.

Prior to 3DPT positioning, optical trackers were installed on both the CT frame and 3DPT fixation needles. After patient setup, CT scans were performed, and the image data were transferred to the INS for real-time guidance.

**Fig. 2:**
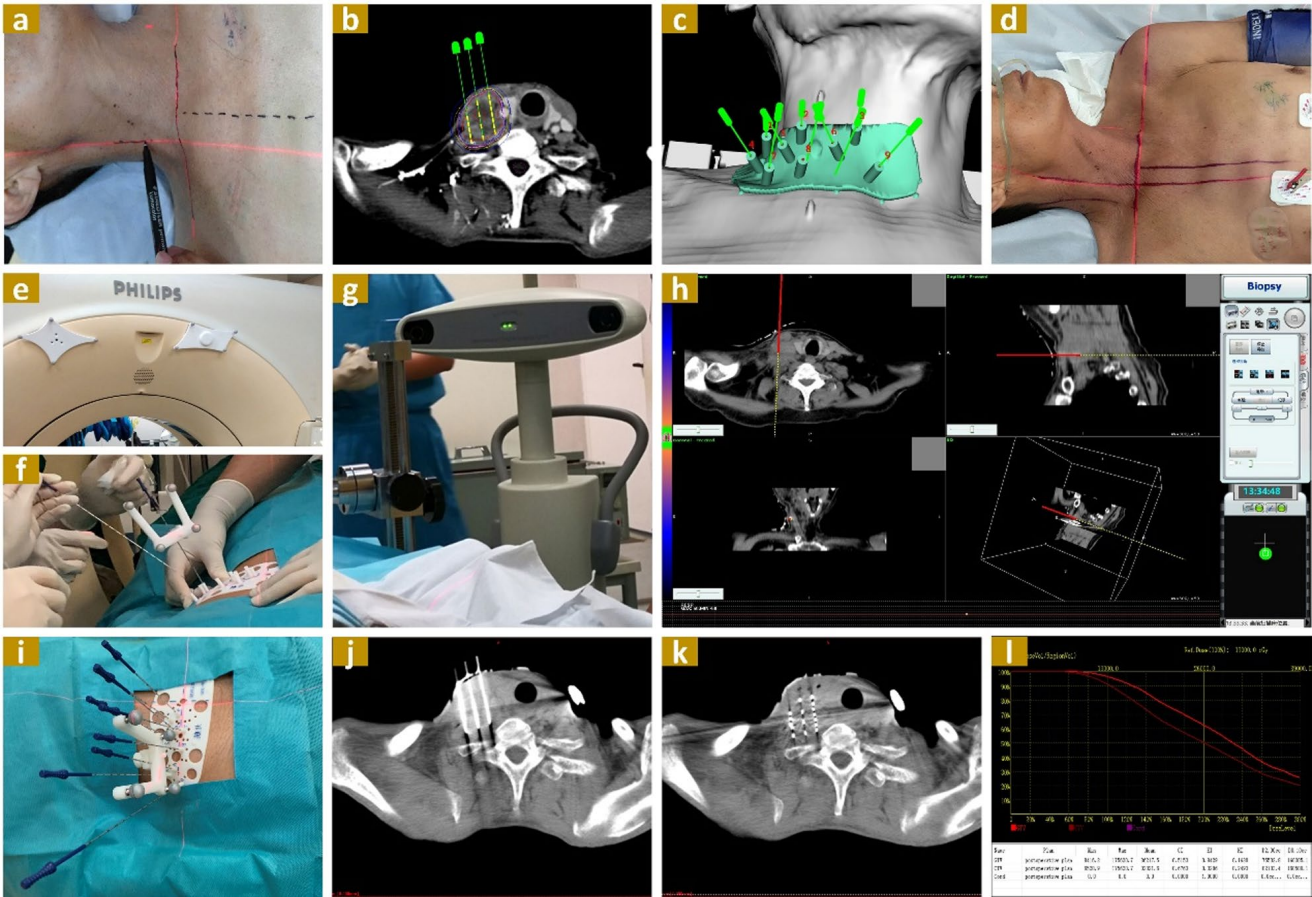
The Procedure of Radioactive Iodine-125 Seed Brachytherapy: **a** CT Simulation: Patient positioning and CT scanning; **b** Preoperative Planning: Target volume delineation and determination of needle and seed placement; **c** 3DPT Production: Construction and printing of 3DPT; **d** 3DPT Alignment: Patient positioning based on original setup marks; **e**-**f **3DPT Alignment (with INS integration): Installation of optical trackers on CT frame and fixation needles; **f** Fixation Needle Insertion: Insertion under 3DPT (or 3DPT + INS) guidance; **g**-**h** Fixation Needle Insertion (with INS integration): Virtual display of needle path position via INS; **i**-**j** Needle Insertion: Remaining needles inserted through 3DPT guide holes; **k** Seed Implantation: Seeds implanted according to preoperative plan; **l** Dose Validation: Assessment of actual dose delivery to target volume. (INS: Image Navigation System; 3DPT: 3D Printing Template)

During 3DPT alignment, the fixation needles were inserted under the guidance of virtual navigation needles displayed in the system interface. The needle positions were adjusted in real-time to ensure alignment between the virtual needles in the intraoperative imaging and the planned positions from the preoperative design (Fig. 2).

### Treatment parameters

1. Puncture information: In the BTPS, the images taken after the intraoperative fixation needle was in place were fused with the preoperative plan images. In the fusion image, both the planned and actual puncture needles were displayed. The angle and depth of the needles before and during the operation were compared, and the absolute value of the difference was taken as the error. Meanwhile, the straight-line distances between the two needles tip positions were recorded.
2. Dosimetry information: In the BTPS, every dosimetry parameter from the preoperative and postoperative plans was compared, including GTV D90, D100 (the dose of 100% GTV received), V100, V150 (volume fraction of 150% prescribed dose that GTV received), V200 (volume fraction of 200% prescribed dose that GTV received). Conformal index (CI) [9], CI=(VT, ref/VT) × (VT, ref/Vref), where VT, VT, ref, and Vref are the volume of the target area, the volume of the target area receiving the prescribed dose, and the total volume (cm3) contained in the prescribed dose, respectively. The optimal CI was 1, which indicated that the prescribed dose covered the target area but the received dose outside the target area was lower than the prescribed dose. A larger CI indicated that the volume inside the target area receiving the prescribed dose was larger, whereas the volume outside the target area receiving the prescribed dose was smaller. External index (EI) [10], EI = (Vref – VT,ref)/VT × 100%. The optimal EI was 0, which suggested that the dose tissues outside the target area received less than the prescribed dose. A higher EI implied that the volume of the prescribed dose received outside the target area was larger. Homogeneity index (HI) [10], HI = (VT,ref ¬– VT,1.5ref)/VT,ref × 100%, where VT,1.5ref is the volume (cm3) of the target area receiving 150% of the prescribed dose. The ideal optimal HI was 100%. A higher HI suggested a more uniform dose distribution in the target area. As the location of the lesions was scattered and the adjacent OAR varied, this study was not designed to compare OAR doses.
3. Other treatment information: In the BTPS, other preoperative and postoperative treatment parameters were collected and compared, including GTV volume and the numbers of needles and seeds.

### Follow up of the treatment outcomes

CT scan was used to detect tumor size changes during follow-up. The International Response Evaluation Criteria in Solid Tumor (RECIST) were used to evaluate treatment response [11]. Complete response was defined as complete disappearance of the target tumor. Partial response was defined as decrease of target lesion diameter to ≤ 30% of that at baseline. Progressive disease was defined as target lesion diameter increase by ≥ 20% or the appearance of new lesions. Stable disease was defined as any change intermediate between partial response and progressive disease. Puncture complications and radiation-related adverse reactions were graded according to Common Terminology Criteria for Adverse Events (CTCAE) version 5.0 [12]; there were five grades, as follows: minor/grade 1 (no symptoms and no treatment required), moderate/grade 2 (symptoms present and treatment required), severe/grade 3 (symptoms not controlled by drugs, and instrumentation or invasive procedure required), life-threatening/grade 4 (emergency treatment required), and death/grade 5. The main evaluation index was the local control rate (LC). The secondary evaluation indices were overall survival rate (OS) and adverse events (AEs).

### Statistical methods

SPSS 25.0 (IBM Corp., Armonk, NY, USA) was used for statistical analysis. Propensity Score Matching (1:1) was used, with a caliper width equal to 0.2 of the standard deviation. We used absolute standardized differences (ASD) to assess the degree of balance in the baseline covariates between the matched groups. An ASD of ≤ 10% denotes a high degree of balance [13]. T-test and chi-square test were used for measurement data and counting data, respectively when two groups were compared. The Kaplan–Meier method and Log-rank test were used to calculate and compare the LC and OS of the two groups. *P* ≤ 0.05 was considered statistically significant.

## Results

**Fig. 3:**
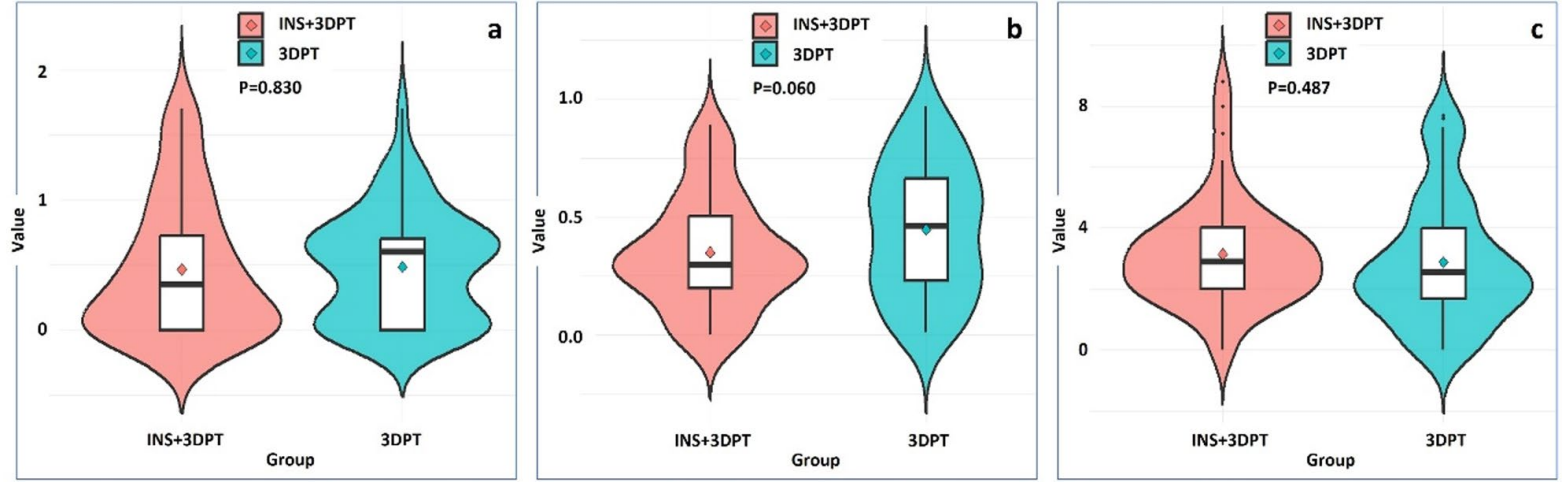
Violin plots of needle insertion errors: **a** Needle angle error: There was no significant difference between the INS+3DPT group and the 3DPT group; **b** Needle depth error: There was no significant difference between the INS+3DPT group and the 3DPT group; **c** Needle tip error: There was no significant difference between the INS+3DPT group and the 3DPT group

After a 1:1 PSM, 27 patients with 3DPT assisted CT guided RSBT were identified. The tumor types of 54 patients included: head and neck tumors (18 cases), cervical cancer (11 cases), colorectal cancer (9 cases), lung cancer (3 cases), esophageal cancer (3 cases), breast cancer (3 cases), sarcoma (2 cases), ovarian cancer (2 cases), prostate cancer (1 case), chordoma (1 case), and invasive fibroma (1 case). Most patients received RSBT as salvage therapy for local recurrence after previous surgery or radiotherapy (49 cases), while some underwent RSBT as primary treatment after declining conventional options (5 cases). Systemic therapy regimens varied significantly among patients both before and after RSBT treatment. The baseline characteristics of the two groups of patients showed good consistency (Table 1). 52 and 54 needles were measured in INS+3DPT group and 3DPT group, respectively. The needle errors of two groups were relatively small, with needle angle error < 1 degree, needle depth error and needle tip error <5 mm. There was no statistical difference between the two groups (*p* > 0.05) (Table 2; Fig. 3).

**Table 1:** Patient characteristics before and after PSM

Characteristics	INS+3DPT (*n* = 27)	Before PSM			After PSM		
		3DPT (*n* = 130)	*p*	ASD	3DPT (*n* = 27)	*p*	ASD
Gender			0.426	0.171		0.780	0.076
Male	17 (63%)	71 (54.6%)			16 (59.3%)		
Female	10 (37%)	59 (45.4%)			11 (40.7%)		
Age (years)	59.7 ± 12.03	58.4 ± 13.66	0.647	0.101	59.9 ± 11.14	0.944	0.020
KPS	80 (60–90)	80 (60–100)	0.921	0.021	80 (70–90)	0.862	0.048
Treatment site			0.002	0.538		0.873	0.076
H&N	13 (48.2%)	20 (15.4%)			11 (40.7%)		
CW	1 (3.7%)	16 (12.3%)			2 (7.5%)		
PC	11 (40.7%)	62 (47.7%)			11 (40.7%)		
RR	2 (7.4%)	11 (8.5%)			3 (11.1%)		
Lesion volume (cm^3^)	41.3 ± 27.90	36.5 ± 32.44	0.48	0.150	42.3 ± 18.54	0.880	0.042

*H&N* Head and Neck, *CW* Chest Wall, *PC* Pelvic Cavity, RR Retroperitoneal Region, *ASD* Absolute Standardized Difference

**Table 2:** Comparison results of preoperative and intraoperative needle angles and depths

Parameters	INS+3DPT (*n* = 52)	3DPT (*n* = 54)	*p*
Needle angle error	0.5 ± 0.52	0.5 ± 0.42	0.830
Needle depth error	3.5 ± 2.38	4.4 ± 2.80	0.060
Needle tip error	3.1 ± 1.75	2.9 ± 2.06	0.487

The mean activity of seed used in the INS+3DPT group and 3DPT group was 0.5mCi with the mean number of seed used was 61, and the mean number of needle used were 15 and 16, respectively. The values of various dosimetric parameters between the two groups were similar, and the differences were not statistically significant (*P* > 0.05) (Table 3).

**Table 3:** Comparison results of postoperative plan parameters

Parameters	INS+3DPT (*n* = 27)	3DPT (*n* = 27)	*p*
Seed activity	0.5 ± 0.06	0.5 ± 0.05	0.981
Needle number	15 ± 7	16 ± 7	0.414
Seed number	61 ± 24	61 ± 16	0.880
D90	138.2 ± 15.07	134.5 ± 10.46	0.312
D100	74.1 ± 17.08	75.9 ± 13.30	0.665
V100	93.4 ± 3.10	92.8 ± 2.56	0.398
V150	70.0 ± 14.61	69.8 ± 12.36	0.951
V200	41.3 ± 15.92	41.8 ± 11.34	0.899
CI	0.6 ± 0.16	0.5 ± 0.17	0.101
EI	0.5 ± 0.38	0.5 ± 0.29	0.957
HI	0.3 ± 0.14	0.3 ± 0.13	0.956

**Fig. 4:**
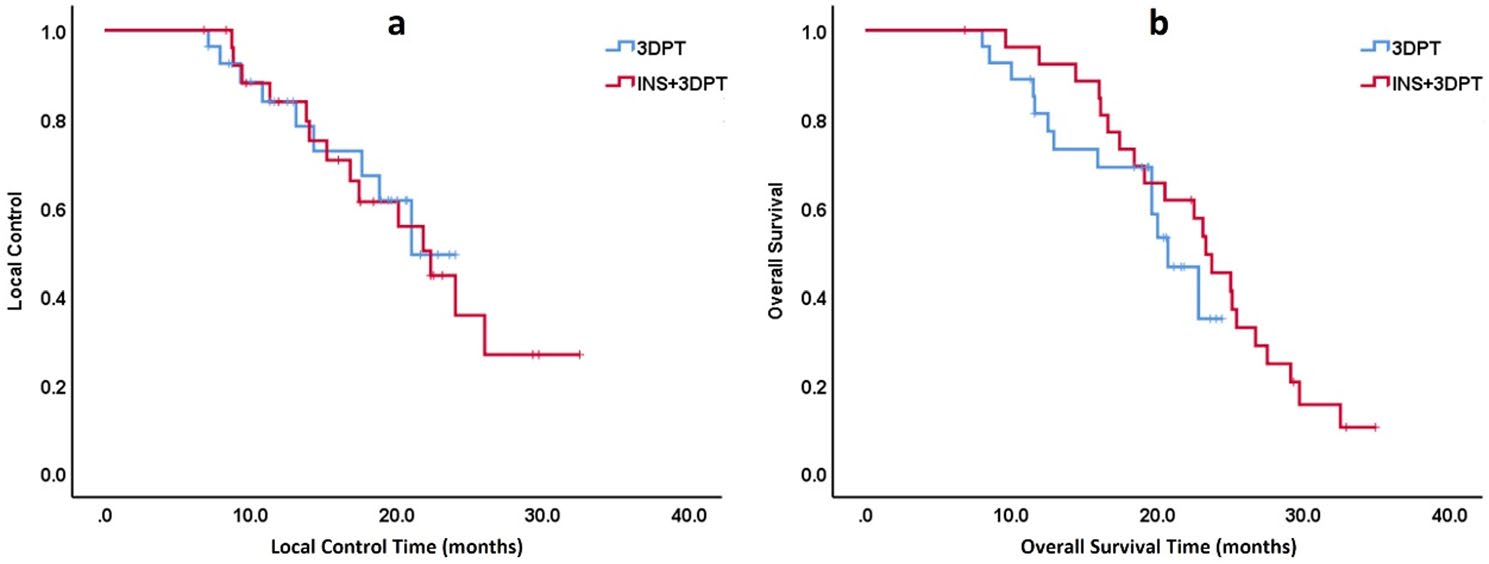
The treatment outcomes of the two groups: **a** The local control results were similar between the INS+3DPT group and the 3DPT group; **b** The survival outcomes were similar between the INS+3DPT group and the 3DPT group

The median follow-up time for the entire group was 20.2 (6.8–34.9) months, with 23.1 (6.8–34.9) months for the INS+3DPT group and 19.4 (8.0-24.4) months for the 3DPT group, respectively. A total of 22 patients in the INS+3DPT group died and 14 cases experienced local failure; A total of 13 patients in the 3DPT group died and 9 cases experienced local failure. The 1-year and 2-year LC in the INS+3DPT group were 83.8% and 35.6%, respectively, while the 1-year and 2-year LC in the 3DPT group were 83.8% and 49.2%, respectively. There was no statistically significant difference between the two groups (*p* > 0.05); The 1-year and 2-year OS in the INS+3DPT group were 92.3% and 45.1%, respectively, while the 1-year and 2-year OS in the 3DPT group were 81.2% and 34.8%, respectively. There was no statistically significant difference between the two groups (*p* > 0.05) (Fig. 4). One case of skin ulceration occurred in the 3DPT group, and there were no ≥ Level 3 adverse events.

## Discussion

3DPT can effectively control seed needles, and several studies have shown that it has good accuracy with small needle path error and postoperative dose can meet the requirements of preoperative plan [3, 8, 14]. INS, on the other hand, is another solution used for puncture intervention. This technology is achieved by fusing and registering preoperative CT, MRI, or PET images with intraoperative images. During operation, a tracker (optical or magnetic positioning) tracks the location of the puncture needle on the fusion image to guide the operating doctor in puncture in real-time [15]. It is increasingly used in puncture related procedures such as biopsy and ablation [16–18]. However, there is no relevant data on whether combining the INS and 3DPT for RSBT is better than simply applying 3DPT.

In this study, the baseline ADS of both groups was < 10% after 1:1 matching, indicating good balance. For INS combined with 3DPT group, optical navigation was used. Due to the large volume of the tracker, it cannot be installed on all seed needles, so only the fixation needles were tracked. In previous study, it was suggested that feasible and accuracy of RSBT assisted by INS combined with 3DPT is good. The mean errors of needle angle, needle depth, and needle tip are 0.5 degrees, 4.0 mm, and 3.1 mm, respectively. The postoperative dose can meet the requirements of preoperative planning [4]. This study added a comparison with simple 3DPT data. In terms of needle path error, the mean needle angle error of both groups was 0.5 degrees, the mean needle depth errors were 3.5 mm and 4.4 mm, and the mean needle tip errors were 3.1 mm and 2.9 mm, respectively. It can be seen that whether using INS combined with 3DPT or simply using 3DPT, both groups have good needle insertion accuracy.

Previous studies on the accuracy of needle paths of 3DPT have shown that for chest lesions, the mean errors of needle angle, needle depth, and needle tip were 2.8 degrees, 5.2 mm, and 4.4 mm, respectively [19]; For retroperitoneal lesions, the mean errors of needle angle, needle depth, and needle tip were 2.7 degrees, 6.9 mm, and 4.5 mm, respectively [20]; For pelvic lesions, the mean errors of needle angle, needle depth, and needle tip were 2.2 degrees, 8 mm, and 4 mm, respectively [21]. The error of needle path of 3DPT group in this study is slightly smaller than that of the previous studies, and it is considered to be related to a higher number of cases in the head/ neck and chest wall (48.1%). Due to the affect of needle depth and the accuracy of template alignment, the error in the head/ neck and chest wall region is often smaller than that in the pelvic/ retroperitoneal region [4].

The dosimetric validation studies on the combination of 3DPT and INS and the 3DPT alone have shown that postoperative doses in various parts of the body can meet the requirements of the preoperative plan well [3, 4]. There is no relevant study comparing the therapeutic effects of the two. This study showed that there was no significant difference (*p* > 0.05) in the local control rate (2-year local control rate of 35.6% and 49.2%, respectively) and overall survival (2-year survival rate of 45.1% and 34.8%, respectively) between the INS combined with 3DPT group and the simple 3DPT group which considering the good consistency of doses between the two groups, that is the consistency of doses maybe translate into consistency of efficacy. Due to the survival is still related to various factors such as the type of primary tumor, tumor staging and general condition of the patient, the evaluation of the efficacy of RSBT often focuses more on the local control.

According to the COMIRI (COMplexity Index of interventional Radiotherapy Implants) classification system [22], both INS+3DPT and 3DPT alone are considered very high-complexity interventions (overall score ≥ 11). While both approaches fall under similar complexity classifications, the workflow of 3DPT alone is relatively more straightforward in practice. This streamlined process, combined with our findings of comparable therapeutic outcomes, suggests that 3DPT alone may be the more practical approach in current clinical settings.

Beyond our extracranial cohort, the clinical application of 3D printing–assisted brachytherapy continues to expand to intracranial tumors, particularly brain metastases in the reirradiation setting. Recent systematic reviews demonstrate that stereotactic brachytherapy achieves high 1-year local control rates (93.3%–100%) with low toxicity and no ≥Grade 3 acute complications for brain metastases [23]. This expansion is further supported by recent clinical guidance, with American Society for Radiation Oncology (ASTRO) conditionally recommending various reirradiation techniques, including brachytherapy, for WHO grade 4 diffuse glioma recurrence [24]. The stereotactic implantation workflow using 3D printing templates shares fundamental principles across anatomical sites, making our comparative findings relevant beyond the extracranial applications studied. However, such complex interventional procedures should be performed in highly specialized centers with multidisciplinary collaboration between radiation oncologists, neurosurgeons, and medical physicists, as recognized by the COMIRI classification system’s designation of these interventions as very high complexity.

The limitations of this study include: (1) significant heterogeneity in patient characteristics - most cases were salvage treatments for post-surgery or post-radiation recurrence, while others were primary treatments for patients who declined conventional options. The varying systemic therapy regimens before and after RSBT could substantially impact clinical outcomes. Therefore, this study primarily focused on analyzing technical parameters (needle path accuracy and dosimetric parameters), with clinical outcomes serving only as preliminary observations. Future studies with larger sample sizes will be needed to properly analyze factors affecting clinical efficacy; (2) due to the scattered location of tumor sites and adjacent OAR, and the low incidence of treatment related AEs, it is not yet possible to effectively calculate and analyze the dose distribution of OAR and its relationship with toxicity; (3) INS combined with 3DPT does not show advantages and the process is more complex than that of 3DPT. If a more optimized combination method is explored, there may be different results. Further case accumulation and in-depth research are needed.

## Conclusion

Both INS combined with 3DPT and 3DPT alone have good therapeutic accuracy. There was no significant difference between the two in terms of needle accuracy, dosimetric accuracy, and clinical efficacy. At present, the use of 3DPT alone in clinical practice can achieve good therapeutic quality. Future research should optimize and improve technical methods, increase sample size, and lengthen the follow-up period in order to more accurately delineate the differences in efficacy and safety between the two.

## Data Availability

The data that support the findings of this study are available from the corresponding author, J.W., upon reasonable request. Restrictions apply to the availability of these data, which were used under license for this study.
